# Evaluating Comparative Effectiveness Research Priorities for Care Coordination in Chronic Obstructive Pulmonary Disease: A Community-Based eDelphi Study

**DOI:** 10.2196/resprot.4591

**Published:** 2015-08-12

**Authors:** Michael Stellefson, Julia Alber, Samantha Paige, Daniela Castro, Briana Singh

**Affiliations:** ^1^ Center for Digital Health and Wellness Department of Health Education and Behavior University of Florida Gainesville, FL United States; ^2^ Center for Heath Behavior Research Department of Biostatistics and Epidemiology University of Pennsylvania Philadelphia, PA United States; ^3^ Center for Digital Health and Wellness Department of Applied Physiology and Kinesiology University of Florida Gainesville, FL United States; ^4^ Center for Digital Health and Wellness College of Public Health and Health Professions University of Florida Gainesville, FL United States

**Keywords:** capacity building, community engagement, comparative effectiveness research, Internet, patient-centered care, patient-centered outcomes research, pulmonary disease, Web-based collaboration

## Abstract

**Background:**

Despite research supporting the use of care coordination in chronic obstructive pulmonary disease (COPD), there is relatively little known about the comparative effectiveness of different strategies used to organize care for patients. To investigate the most important COPD care coordination strategies, community-based stakeholder input is needed, especially from medically underserved populations. Web-based platforms are electronic tools now being used to bring together individuals from underrepresented populations to share input and obtain clarification on comparative effectiveness research (CER) ideas, questions, and hypotheses.

**Objective:**

Use low computer-literate, collaborative survey technology to evaluate stakeholder priorities for CER in COPD care coordination.

**Methods:**

A mixed-method, concurrent triangulation design was used to collect survey data from a virtual advisory board of community-based stakeholders including medically underserved patients with COPD, informal caregivers, clinicians, and research scientists. The eDelphi method was used to conduct 3 iterative rounds of Web-based surveys. In the first 2 survey rounds, panelists viewed a series of “mini research prospectus” YouTube video presentations and rated their level of agreement with the importance of 10 COPD care coordination topics using 7-point Likert scales. In the final third-round survey, panelists ranked (1=most important, 8=least important) and commented on 8 remaining topics that panelists favored most throughout the first 2 survey rounds. Following the third-round survey, panelists were asked to provide feedback on the potential impact of a Web-based stakeholder engagement network dedicated to improving CER in COPD.

**Results:**

Thirty-seven panelists rated the following care coordination topics as most important (lower means indicate greater importance): (1) measurement of quality of care (mean 2.73, SD 1.95); (2) management of COPD with other chronic health issues (mean 2.92, SD 1.67); (3) pulmonary rehabilitation as a model for care (mean 3.72; SD 1.93); (4) quality of care coordination (mean 4.12, SD 2.41); and (5) comprehensive COPD patient education (mean 4.27, SD 2.38). Stakeholder comments on the relative importance of these care coordination topics primarily addressed the importance of comparing strategies for COPD symptom management and evaluating new methods for patient-provider communication. Approximately one half of the virtual panel assembled indicated that a Web-based stakeholder engagement network could enable more online community meetings (n=19/37, 51%) and facilitate more opportunities to suggest, comment on, and vote for new CER ideas in COPD (n=18/37, 49%).

**Conclusions:**

Members of this unique virtual advisory board engaged in a structured Web-based communication process that identified the most important community-specific COPD care coordination research topics and questions. Findings from this study support the need for more CER that evaluates quality of care measures used to assess the delivery of treatments and interventions among medically underserved patients with COPD.

## Introduction

### Background

Chronic obstructive pulmonary disease (COPD), a chronic lung condition characterized by progressive airflow limitation, shortness of breath, and productive cough, is the third leading cause of death in the United States [1]. Estimates indicate that approximately 15 million US adults are living with COPD [2]; however, the actual number of adults living with COPD is likely much higher, because COPD often goes undetected and undiagnosed [2]. In 2010, the economic burden of COPD in the United States was approximately US$ 50 billion, including US$ 30 billion in direct and US$ 20 billion in indirect health care costs [3]. The largest share of the cost burden was caused by poorly managed comorbidities (eg, hypertension, heart disease, and mental illness) experienced by patients [4]. Although different approaches to managing COPD and its complicating factors have been studied for decades [5], so far relatively little is known about the comparative benefits and harms of care coordination strategies used to help patients manage COPD in the presence of morbidity and comorbidity [6]. Care coordination has been defined as, “the deliberate organization of patient care activities between 2 or more participants (including the patient) involved in a patient's care to facilitate the appropriate delivery of health care services” [7]. Organizing care involves health care personnel and resources needed to carry out patient care, which is often governed by information exchange among participants responsible for different facets of care [8]. Participants in the care coordination process can include patients, family caregivers, physicians, nurses, pharmacists, social workers, and others [7].

Inadequate coordination between primary and specialist care and between community and medical settings may be the main reason for poorly managed symptoms observed in patients with long-term conditions such as COPD [9]. Therefore, there are a variety of care coordination strategies used to support self-management and treatment services for patients with COPD, such as smoking cessation, influenza and pneumonia vaccination, pulmonary rehabilitation, and symptomatic and maintenance pharmacotherapies. Effective care coordination in COPD through ongoing multifactorial management can not only reduce health care costs stemming from the effects of poorly managed COPD, such as dyspnea (ie, shortness of breath) exacerbations, but also improve a patient’s health-related quality of life [10]. The most effective chronic care coordination strategies can be identified through comparative effectiveness research (CER), which explores the extent to which the organization of care and communication between health care providers is collaborative and productive [4,8,10,11,12]. In CER, the benefits and harms of alternative treatments and interventions are directly compared to determine their efficacy to prevent, diagnose, treat, and monitor clinical conditions in real-world settings [13]. To ensure that care coordination strategies provide the most benefits to patients with the least potential for harm [14,15], it is recommended that key stakeholders remain engaged in all stages of health care decision-making processes [4,11,12,14,16]. In health care, a “stakeholder” is defined as anyone who can have a significant influence on an organization’s ability to address a health-related issue of interest [14]. Often, health issues such as the presence or absence of a particular disease or condition bring stakeholders from different groups together. These different groups include health care providers, allied health care professionals, patients, and informal caregivers. “Stakeholder engagement” is defined as the process of working collaboratively with and through diverse groups of people to gather input from all those affected by the topic of interest, to help solve the health problem or improve health status of patients [14,17].

To date, there has been limited involvement from individuals most affected by care coordination processes (ie, patients and informal caregivers) in defining CER topics and evaluating COPD care coordination strategies [16,18]. Pickard and colleagues [19] first used 2 phases of in-person meetings with clinicians, researchers, and representatives from health care plans, patient advocacy groups, and professional health organizations to identify “the effectiveness of supplemental oxygen for COPD” as the highest priority CER topic in COPD. Krishnan and colleagues [20] later assembled 54 stakeholders representing government entities, research institutions, health plans, and patient advocacy organizations to develop an updated list of high-priority CER topics in COPD. A series of 2-year workshops sponsored by the COPD Outcomes-Based Network for Clinical Effectiveness and Research Translation explored priorities in the following 4 discrete areas: (1) chronic care; (2) care coordination; (3) acute care; and (4) transitions in care. Within the “care coordination” category, the topics, “management of COPD in the presence of comorbidity” and “pulmonary rehabilitation as a model for care coordination,” were ranked as the 2 highest priorities; however, neither topic was rated as “most important” by participating stakeholders. Although both studies [19,20] identified COPD care coordination priorities for CER, there was limited involvement from actual patients with COPD or their informal caregivers (ie, family members and friends who aid and supervise the daily care of people living with an illness or disability). Stakeholders from these highly relevant groups can help identify important care coordination topics that clinical, administrative, and research-oriented stakeholders may overlook. Supporting frequent collaboration between researchers and key COPD stakeholder groups is essential to sustaining patient-centered research targeted at improving the management and treatment of the disease [18]. Research teams that engage stakeholder groups from underrepresented patient and informal caregiver groups can establish patient-centered priorities for care coordination in COPD through meaningful and collaborative partnerships.

### Current Barriers to Stakeholder Engagement

Current barriers to meaningful stakeholder engagement include lack of interest, limited stakeholder education about research topics and health issues, and human/fiscal resource constraints. These barriers can preclude researchers from identifying, engaging, and interacting with stakeholders; however, there are a number of innovative, technology-based methods that help overcome some of these obstacles [14]. For example, low-cost, Web-based collaborative platforms use convenient virtual spaces (eg, discussion boards, chat rooms, community forums) to decrease the geographical and temporal barriers to recruiting and retaining low socioeconomic status (SES) chronic disease patients. Moreover, diverse patients with ethnic and minority backgrounds are interested in adopting health information and communication technologies (ICTs) that were previously viewed as out of reach for these historically marginalized populations [21,22]. Patients with COPD use the Internet to locate information to self-manage their condition and communicate with others about their health [23]. To improve stakeholder engagement in CER related to COPD, a flexible research infrastructure must exist that is sensitive to sociotechnical changes in collaboration [24]. The flexibility and convenience of the Internet provides a platform to quickly disseminate surveys and anonymous responses to all panelists [25]. Web-based community building enables virtual advisory boards to grow quickly and inexpensively. On Web-based community platforms, stakeholders are given the freedom to securely share ideas, request feedback, and obtain clarification on research questions and hypotheses without having to attend panel meetings at fixed times in fixed locations. Increasing the adoption of Web-based collaborative platforms among low-income, medically underserved patients with COPD will likely improve stakeholder engagement during the research agenda-setting process, which, in turn, can enhance the overall relevance of CER for patients living with COPD [26].

Currently, more CER is needed to help better understand the relative benefits and harms of the many available strategies used to coordinate the delivery of care in COPD. Community-level input on the importance of different strategies can improve the design of CER studies of COPD care coordination approaches in different health care settings. As such, the primary objective of this stakeholder engagement study was to systematically prioritize CER topics related to COPD care coordination using electronic feedback provided by a diverse community-based panel of stakeholders. A secondary objective was to gather stakeholder input that could help inform the development of a Web-based stakeholder engagement network dedicated to evaluating, translating, and disseminating CER findings in COPD care coordination.

## Methods

### Research Design

A mixed-method concurrent triangulation design [27,28] was used to collect both quantitative and qualitative data on the relative importance of different approaches to COPD care coordination. This pragmatic design merged quantitative and qualitative data to generate a greater understanding about the highest priority COPD care coordination topics as perceived by a virtual advisory board of stakeholders living and working in the community. The eDelphi method, an electronic version of the Rand Corporation’s Delphi method [29], was used obtain an informed consensus from stakeholders living and working in or near the university community. The eDelphi method is a group communication process that uses a series of surveys administered to an anonymous, informed panel to achieve convergence of opinions on a particular topic. During an eDelphi study, each panelist is encouraged to form an opinion, and independently reassess his/her opinion based on anonymous feedback from fellow panelists in successive survey rounds [30-32]. Researchers who use the eDelphi method for opinion pooling create a nonconfrontational environment where panelists can potentially modify their opinions with limited peer pressure [33,34]. Web-based survey iteration generally continues for a designated number of rounds until consensus (ie, when agreement exists among at least three fourths of panelists) is reached [33]. Research suggests that completing a structured series of 3 Web-based questionnaires is generally sufficient for reaching consensus [35-38]. Often, panelists are also provided the opportunity to submit qualitative feedback to clarify and expand upon their quantitative rankings of the topic(s) at hand. Researchers are able to corroborate and validate results from an eDelphi study by directly illustrating quantitative data with qualitative findings [39].

Compared with the traditional paper-based and snail mail Delphi, the eDelphi offers researchers several other advantages, including the following: (1) Web-based storage, processing, and transmission of secure data; (2) protection of respondent anonymity; (3) rapid feedback to panelists in the form of tables, charts, and statistics; and (4) fewer logistical challenges generally associated with bringing groups of people together for research-related purposes [25,40,41]. The eDelphi method is substantially different from in-person focus groups and online discussion forums. This consensus-building method provides the opportunity for panelists to provide independent feedback without being in direct communication with others. With anonymous feedback, there is little risk for panelists higher up on professional hierarchies to manipulate the opinions of other panelists [42].

### Panelist Recruitment and Identification

#### Overview

The commitment of stakeholders to participate in eDelphi studies is often related to their level of interest with the topic [30]. In this study, a diverse panel of stakeholders with an interest in COPD care coordination was recruited through a collaboration with a community engagement and research (CEnR) program operating at a large, research-intensive university in the Southeastern United States. The mission of the CEnR program is to reduce health care and research disparities by linking the medically underserved to research opportunities and medical and social services. Partnerships with community organizations and regional agencies provide guidance on appropriate and culturally sensitive collaborative opportunities for participants and researchers. The program serves several primary purposes in the community, including the following: (1) assessing health concerns from the viewpoint of community members; (2) facilitating 2-way communication with the community on how to promote health; (3) linking community members to opportunities to participate in health-related research; and (4) increasing the community’s trust in research through collaboration and partnership. Most community members affiliated with the CEnR program (approximately 1900) belong to low-income, underrepresented minority communities.

#### Patients

Purposive sampling was used to identify patients with COPD registered in a community patient database operated and managed by the CEnR program. All patients were invited to participate by a study navigator working on behalf of the CEnR program. Patients were asked predetermined questions over the telephone to determine whether they met the following inclusion criteria: (1) diagnosis of COPD by a physician or specialist; (2) valid email address on file in the community patient database; (3) minimum 40 years of age; and (4) speak English as a first language. The minimum age of 40 was established because individuals at this age generally begin experiencing COPD symptoms such as excess mucus buildup, wheezing, and productive cough [43]. If patients met inclusion criteria, the study navigator asked whether or not the patient was interested in learning more about the study.

#### Informal Caregivers

Informal caregivers of patients with COPD were recruited using snowball sampling methods that capitalized on patient referrals. Snowball sampling is particularly useful when identifying and recruiting racial/ethnic minorities and individuals with low SES to participate in research [44]. Snowball sampling allows participants from these hard-to-reach populations to act as gatekeepers for recruiting others they know into a study. To identify informal caregivers of medically underserved patients with COPD in this study, patients were asked to refer a maximum of 3 informal caregivers into the study. An “informal caregiver” was defined as someone who met the following criteria: (1) family member or friend of the COPD patient; (2) at least 21 years old; and (3) responsible for helping to provide nonclinical care (eg, medication reminders, self-management support) to at least one COPD patient. The informal caregiver(s) was then provided with the contact information of a research navigator affiliated with the CEnR program, who was available to provide additional information about study enrollment.

#### Clinicians and Research Scientists

Practicing clinicians and research scientists with professional experience studying COPD care coordination were identified through searching VIVO, an intrainstitutional semantic-Web-networking platform used to foster cross-disciplinary team science in the university where the research took place [45]. The VIVO platform uses an interactive database including a variety of scholarly variables (eg, home department/college/laboratory, number and type of grant awards, courses taught, curricula vitae) describing employed clinicians and research scientists. In this study, VIVO was queried by 2 members (MS and JA) of the research team using the following keywords: “chronic obstructive pulmonary disease,” “pulmonary health (adults),” “pulmonary rehab,” “respiratory therapy,” “respiratory health,” “care coordination,” “comparative effectiveness research,” “lung disease,” and “oxygen therapy.” All Web profiles and curricula vitae of identified research scientists and clinicians were evaluated to determine their level of involvement in COPD care coordination research. Clinicians (eg, doctors, physiotherapists, respiratory therapists, nurses) were required to be employed by a university-based clinic or health care facility for a minimum of 5 years, and research scientists were required to be current university faculty investigating at least one aspect of COPD care coordination (eg, disease management, pulmonary rehabilitation, case management, risk management) [20]. Evidence of experience investigating one or more care coordination topics was identified through reviewing grant award activity, publications, and professional presentations.

### Procedures

**Figure 1:**
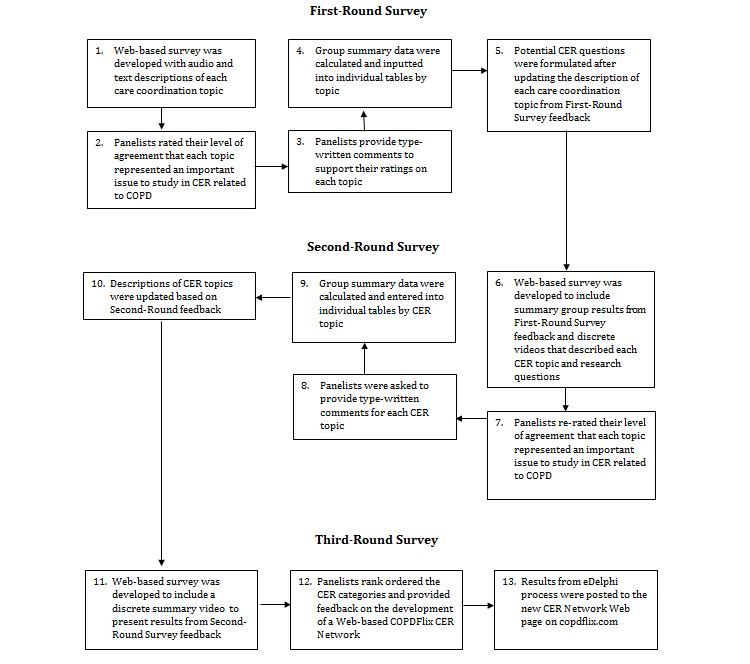
Three-round eDelphi process.

Figure 1 depicts the sequence of 3 iterative rounds of Web-based surveys carried out over the 6-week study period. Interested panelists from each group were sent formal email invitations with an embedded Qualtrics survey hyperlink. After clicking on the hyperlink, panelists were presented with an electronic informed consent document, which provided a description of the study’s purpose and procedures. Panelists were provided with the following information: (1) title of the study; (2) purpose of the study; (3) description of what would be asked of them (ie, completing 3 Web-based surveys over a period of approximately 2-3 months); and (4) description of an US$ 45 gift certificate incentive offered for their participation. Panelists were also made aware that their participation was voluntary. Information on the confidentiality of responses was also provided, including information on Qualtrics password protection, server security, and firewall protections. Panelists were made aware that a security breach of their online data was unlikely and would not likely result in adverse consequences. After providing consent, panelists were directed to the first-round survey.

#### First-Round Survey

In the first-round survey, panelists were provided with a brief (3-4 sentences) bulleted description of 10 COPD care coordination topics selected from the literature [20]: (1) management of COPD when other chronic problems are present; (2) pulmonary rehabilitation as model for care in COPD; (3) depression and mental health management in patients with COPD; (4) measurement of quality of care in patients with COPD; (5) quality of care coordination; (6) comprehensive COPD patient education; (7) cost effectiveness of care; (8) case management in COPD; (9) measuring hospitalization risk in patients with COPD; and (10) patient-centered medical home. Table 1 provides definitions of all 10 COPD care coordination topics evaluated by stakeholder panelists. All panelists were provided background information on each topic using literacy-sensitive text and audio narration. All text-based survey content followed recommended principles for developing Web-based consumer health education materials for older adults [46-48]: (1) enlarged text (14-point font) written at or below a 6th grade reading level; (2) short excerpts of text (ie, 1 primary point/sentence); (3) plain language (ie, communication an audience can understand the first time they read or hear it) with an active voice; and (4) sans-serif font (eg, Arial, Calibri) with left justification and no italics.

Panelists’ level of agreement with the importance of each of the 10 COPD care coordination topics was evaluated using a 7-point Likert scale (1=strongly disagree, 7=strongly agree). Panelists were also asked to provide demographic information using items adapted from the 2014 Behavioral Risk Factor Surveillance System’s Questionnaire [49]. Demographic items included the following: age (years), sex (male, female, and other), race (white, black/African American, American Indian/Alaska Native, Asian, Pacific Islander, and other), ethnicity (Hispanic/Latino/Spanish origin and non-Hispanic), highest grade or year completed in school (never attended school, grades 1-8, grades 9-11, grade 12 or general educational development diploma, college 1-3 years, and college 4 years or more), marital status (married, widowed, divorced, separated, and never married), and household income (less than US$ 10,000, less than US$ 15,000, less than US$ 20,000, less than US$ 25,000, less than US$ 35,000, less than US$ 50,000, less than US$ 75,000, and US$ 75,000 or more). Panelists were given up to 2 weeks (August 22, 2014, to September 8, 2014) to complete the first-round survey.

**Table 1:** Definitions of chronic obstructive pulmonary disease care coordination topics evaluated by stakeholder panelists.

Care coordination topic	Definition	Sample comparative effectiveness research question^a^
Management of chronic obstructive pulmonary disease (COPD) when other chronic health problems are present	Participation among multiple health care professionals in the treatment of COPD and other comorbidities experienced by the patient	Would a standard process for identifying chronic health conditions commonly found in patients with COPD help improve a doctor’s ability to treat patients?
Pulmonary rehabilitation as a model for care in COPD	Formal program aimed at improving the quality of life for patients with chronic lung disease that includes a broad range of activities such as nutritional counseling, breathing retraining, exercise training, and practicing energy conservation	Which setting for pulmonary rehabilitation is most effective for different patients with COPD?
Depression and mental health management in patients with COPD	Active screening for depression and the management of mental health provided by trained depression care managers and primary care providers, including antidepressant treatment and/or psychotherapy provided by a supervising psychiatrist	Would patients undergoing regular screening for depression show improvements in mental and physical health outcomes?
Quality of care coordination	Measuring how effectively patient's needs and preferences are understood and communicated at the right time to the right people, and whether or not this information is used to provide safe, appropriate, and effective care to the patient	What factors are associated with high-quality care coordination in patients with COPD?
Measurement of quality of care in patients with COPD	The degree to which various strategies used for managing treatment across health care providers and services produces the desired health outcomes and improves the patient’s quality of life	What should be measured while a patient with COPD is following a prescribed treatment plan?
Comprehensive COPD patient education	Knowledge- and skill-building programs for patients with COPD aimed at improving the patient’s ability to independently self-monitor and manage COPD	What is the comparative effectiveness of different types of educational programs designed to teach patients how to use their respiratory inhaler?
Cost effectiveness of care	The direct comparison of the costs and health benefits of a treatment or intervention option	What is the cost effectiveness of different models of care coordination such as pulmonary rehabilitation versus comprehensive patient education?
Case management in COPD	Strategies that involve working with health care providers to coordinate and monitor treatments to meet individual patient needs	What is the effectiveness of case management programs for patients with other chronic conditions such as heart failure and diabetes, compared with regular care in patients with COPD?
Measuring hospitalization risk in patients with COPD	Evaluating and identifying which patients are at the greatest risk of hospitalization and rehospitalization to inform and modify risk-specific care plans	Is it possible to use a screening tool to identify which patients are at greatest risk of visiting the doctor’s office or emergency room on a more regular basis?
Patient-centered medical home	Care strategy that uses a team of health care providers to monitor and improve patient care in the most accessible, convenient way for a patient	Does an individual patient with COPD experience better outcomes if he/she participates in a patient-centered medical home as compared to following other COPD treatment plans?

^a^Adapted from sample questions suggested by Krishnan and colleagues [20].

#### Second-Round Survey

**Figure 2:**
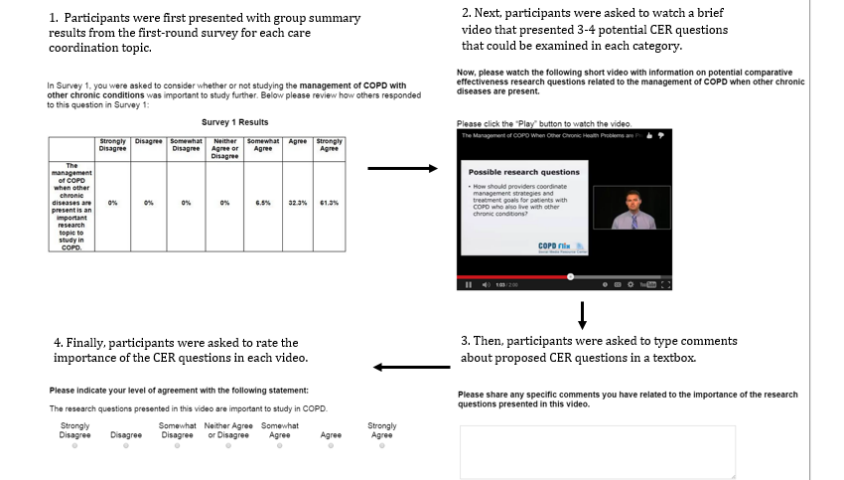
Item sequence during second-round survey administered on Qualtrics.

After the first-round survey closed, 2 members of the research team (MS and JA) compiled all panelist ratings and type-written comments from the first-round survey. These data were used in conjunction with other stakeholder education materials [20] to develop a 1-2-minute video transcript describing each of the 10 COPD care coordination topics. Transcripts were used to record a series of “mini research prospectus” YouTube video presentations delivered by the lead investigator (MS). Figure 2 illustrates how topics were described and how surveys were delivered to panelists during the second-round survey. Each video was uploaded onto the Qualtrics survey interface, and all presentations followed a structured sequence of PowerPoint slides that described (1) contextual information supporting the need to investigate the topic; (2) 3-4 potential CER questions that could be examined within each topic; and (3) a brief “take-home” justification for why each care coordination topic may be important to explore in future CER. PDF versions of video transcripts were made available for users to download and review.

All consenting panelists who successfully completed the first-round survey were sent an email invitation with an embedded hyperlink to access this second-round survey. In the second-round survey, panelists were asked to review summary results from the first-round survey and watch all 10 care coordination YouTube videos developed by the research team using summary data from round one. Following each video, panelists were invited to enter their own comments into a textbox and rate their level of agreement with the importance of CER questions explained on each video (1=strongly disagree, 7=strongly agree). Like in round one, panelists were given 2 weeks to submit their survey responses (September 10, 2014, to September 25, 2014).

#### Third-Round Survey

Following the closure of the second-round survey, 2 researchers compiled all panelists’ ratings and type-written comments from the second-round survey to create a final 6-minute video presentation delivered by the lead investigator. This final video summarized all eDelphi results from the first and second rounds. Specifically, it described the level of panelist convergence around the highest priority care coordination topics from the first to second rounds. The video was embedded onto the Qualtrics survey platform using the YouTube media player. After viewing the final summary video, panelists were asked to rank-order 8 remaining topics that received consensus support in the prior 2 rounds (1=most important, 8=least important). In addition, panelists were asked to comment on why they believed the topic they ranked as “1” was most important to study further.

Panelists were also asked to answer questions related to the potential impact of a Web-based stakeholder engagement network dedicated to fostering and improving CER in COPD. Panelists reported how likely they would be to visit the stakeholder engagement network website on a 6-point Likert scale (1=very unlikely, 6=very likely), and they were asked to indicate how often they would visit the website using a 5-point Likert scale (1=rarely or never, 5=every day or almost every day). Panelists were also asked to rank 5 potential purposes of the website from 1 to 5 (1=most important, 5=least important). Potential purposes of the Web-based stakeholder engagement network included learning from others, accessing research reports online, study recruitment, and building trust. Panelists could also enter in their own desired purpose using a field marked “Other.” Finally, panelists responded to the following open-ended questions: (1) “If you were to join a CER Network website, what roles or responsibilities would you see for yourself?”; (2) “What would you expect to gain from visiting a CER Network website?”; and (3) “What types of resources would you like available on a CER Network website?” Panelists could select from a list of all responses that they felt answered each question. Panelists were given 2 weeks to complete the third-round survey (October 15, 2014, to October 29, 2014), and following completion, each participant was redirected to a secure external website to provide contact information that would enable them to receive a US$ 45 gift card incentive for their participation.

### Data Analysis

Once duplicate surveys were deleted from the final database and a code number was assigned to each survey, the Internet protocol addresses were deleted to eliminate any linkages between participant names and email addresses. Qualitative and quantitative data were analyzed separately and independently. Two researchers scanned all submitted comments from round one and round two surveys to determine whether any panelists reported confusion related to any of the topic categories. Round-by-round panelist response and attrition rates were computed using frequency statistics. Frequency statistics were also computed to report the sociodemographic characteristics of panelists. Panelists’ ratings on each COPD care coordination topic were summarized in each round using mean (SD) statistics to quantify the collective judgment of respondents [28]. Convergence of panelists’ opinions (ie, consensus) was judged to be reached when 80% or more of panelists’ ratings were rated as “6=agree” or “7=strongly agree” on the 7-point Likert scales [50].

To determine which COPD care coordination research topics were elaborated on the most by panelists, Braun and Clarke’s [51] 6-step approach to thematic analysis was completed by 2 members of the research team to evaluate third-round qualitative data describing panelists’ reasons for selecting the most important care coordination topic. These steps included the following: (1) becoming familiar with the data; (2) generating initial codes; (3) searching for themes; (4) reviewing themes; (5) defining and naming themes; and (6) producing a final summary report. Each research team member reviewed transcripts independently, and all panelist comments were analyzed using open coding [52]. A codebook was developed based on open codes that included each code definition [53]. Cohen kappa statistic was calculated for each code to determine intercoder reliability; acceptable kappa statistics were .75 or more [54,55]. When disagreement occurred between coders, a third member of the research team was asked to help resolve the discrepancy. Thirty unique codes showed evidence of adequate intercoder reliability (Table 2). After codes were established, 2 researchers analyzed the qualitative data by applying the codes and grouping them into common themes. Representative comments were identified for each emergent theme.

After analyzing the third-round qualitative data with thematic analysis, both qualitative and quantitative sets of results were merged by directly comparing the quantitative rankings from the third-round survey with the qualitative feedback describing panelists’ reasons for selecting the highest priority COPD care coordination topic to study in future CER. Additional qualitative feedback was used to determine which COPD care coordination topics were elaborated on to the greatest extent. Finally, frequency and descriptive statistics were computed to analyze data on stakeholders’ beliefs regarding the potential impact of a Web-based stakeholder engagement network for advancing CER in COPD.

**Table 2:** Round three survey codes, definitions, and kappa values.

Code	Definition	Kappa
Behaviors/lifestyle	Includes patient health behaviors or lifestyle choices (eg, smoking, healthy eating)	>.99
Comorbidity	Mention of having multiple health problems at once (COPD^a^ and asthma)	>.99
Coordination	Individuals working together or planning together for a common purpose; includes working as a team	.90
Coping	Patient strategies or discussion of ways to deal with health issues caused by or related to COPD	.91
Cost	Refers to money needed or spent on the care, management, or treatment of COPD	>.99
Diagnosis	Diagnosis of COPD or the health issues (eg, diagnosis of depression) stemming from COPD symptoms	.91
Doctor visits	Visits to health care providers that are involved in the treatment of COPD, NOT including emergency room visits	>.99
Patient education	References to the need to learn more or increase understanding about COPD, the treatments of COPD, or anything else related to COPD	.93
Effectiveness	References to the effectiveness of COPD treatments to manage or prevent health issues; include cost effectiveness; comparison benefits versus negative effects	.95
Emergency room visits	Mention of the occurrence or prevention of emergency room visits due to COPD symptoms or complications	>.99
Expectation	References to the patient’s expectation of COPD and its treatment	.80
Follow-up	Mention of the patient or provider following up on a treatment or issue related to COPD	.80
Health effects	Mentions of any health outcome related to COPD or symptoms of COPD (eg, breathing issues)	.94
Home	Reference to the in-home care or treatment of COPD	>.99
Hospital stay	Mention of having to stay at a hospital for a day or more for a treatment related to COPD; hospital visit outside of a regular doctor visit; not including emergency room visits	>.99
Informed	Mention of all individuals involved or affected by COPD having the knowledge to make appropriate or proper decisions about treatment or care	.95
Limitations	Reference to the downfalls or possible cons of a treatment or care strategy for COPD	>.99
Management	General references to managing COPD	.96
Medicine	Mention of any type of medicine taken to treat COPD or health issues related to COPD	>.99
Mental health	Mention of mental health concerns, such as depression, stemming from COPD diagnosis, symptoms, or treatment	>.99
Monitor	Monitoring the progress or effects of COPD symptoms and treatment	>.99
Necessary/helpful	Reference to someone or something being necessary, sufficient, or helpful in the COPD treatment or management	.99
Death	Discussion or reference to passing away	>.99
Patient-provider communication	Reference to the communication or lack thereof between a patient and the health care providers involved in their treatment	>.99
Personal experience	A patient or provider references a specific event that occurred related to diagnosis or treatment of COPD	>.99
Personalization	Reference to the unique factors of patients and the need for individualized treatment, care, or consideration	.96
Prevention	Reference to the strategies and need for preventing COPD or the complications of COPD	>.99
Health-related quality of life	Reference to quality of life, such as the importance or the negative/positive effects that COPD has on quality of life	.86
Standardize	Reference to the standardization of treatment options and management of COPD for patients	.75
Statistics	References to numerical data related to COPD, such as number of deaths caused by COPD or the number of people living with COPD	.80

^a^Chronic obstructive pulmonary disease

## Results

### Panelist Composition

Table 3 describes the demographic characteristics of 37 stakeholder panelists who consented to participate in the study, including patients (n=23), informal caregivers (n=3), clinicians (n=2), researchers (n=6), and unidentified participants (n=3).

**Table 3:** eDelphi panelists’ sociodemographic characteristics (n=37).^a^

Demographic variable		Patients n (%)	Informal caregivers n (%)	Clinicians n (%)	Researchers n (%)	Unidentified^b^ n (%)
**Sex**						
	Female	11 (47.8)	3 (100.0)	0 (0.0)	3 (50.0)	2 (33.3)
	Male	11 (47.8)	0 (0.0)	2 (100.0)	3 (50.0)	1 (16.6)
	Other	1 (4.4)	0 (0.0)	0 (0.0)	0 (0.0)	0 (0.0)
**Race/** **Ethnicity**						
	White	15 (65.2)	3 (100.0)	1 (50.0)	5 (83.3)	2 (33.3)
	Black/African American	6 (26.0)	0 (0.0)	0 (0.0)	0 (0.0)	1 (16.6)
	Hispanic or Latino	3 (13.0)	0 (0.0)	0 (0.0)	0 (0.0)	1 (16.6)
	Other	3 (13.0)	0 (0.0)	1 (50.0)	1 (16.6)	1 (16.6)
**Highest grade completed**						
	Less than a high-school degree	2 (8.7)	0 (0.0)	0 (0.0)	1 (16.6)	2 (8.7)
	High-school/general educational development	5 (21.7)	0 (0.0)	0 (0.0)	0 (0.0)	0 (0.0)
	Some college^c^	16 (69.5)	3 (100.0)	2 (100.0)	6 (100.0)	1 (16.6)
**Household income**						
	<US$ 25,000	18 (78.2)	0 (0.0)	0 (0.0)	0 (0.0)	0 (0.0)
	US$ 25,000-US$ 49,999	4 (17.3)	1 (33.3)	0 (0.0)	0 (0.0)	3 (50.0)
	>US$ 50,000	0 (0.0)	1 (33.3)	2 (100.0)	6 (100.0)	0 (0.0)
**Marital status**						
	Married or widowed	7 (30.4)	1 (33.3)	2 (100.0)	5 (83.3)	2 (33.3)
	Divorced	8 (34.7)	0 (0.0)	0 (0.0)	0 (0.0)	0 (0.0)
	Separated	4 (17.3)	0 (0.0)	0 (0.0)	1 (16.6)	0 (0.0)
	Never married	4 (17.3)	2 (66.6)	0 (0.0)	0 (0.0)	1 (16.6)

^a^Panelists identified with more than 1 stakeholder group (n=3).

^b^Three (n=3) individuals who did not identify their stakeholder role did not provide any demographic information.

^c^Some college is defined as completing at least 1 year of coursework in the college/university setting.

#### Patients

One-hundred and thirty-seven patients living with COPD in the community met eligibility criteria to serve on the virtual advisory board. Fifty-four patients (39%) agreed to participate; however, less than half of those who agreed to participate (n=23) completed the first-round survey. An equal number of female (n=11) and male (n=11) patients joined the panel (mean age 57.65 years, SD 6.80 years). Most patient panelists identified as being white (n=15, 65%), with fewer identifying as black/African American (n=6, 26%). The majority of patient panelists (n=16, 70%) reported completing at least one year of college; yet over 75% of patients (n=18) reported annual household incomes less than US$ 25,000/year. Almost three quarters of patients were either divorced (n=8), separated (n=4), or never married (n=4).

#### Informal Caregivers

Only 3 informal caregivers agreed to join the virtual panel. All informal caregiver panelists were women, white, and reported completing at least one year of college. The mean age of informal caregivers was 41 years (SD 9.54 years). Annual household income for each informal caregiver varied, with a panelist reporting an income less than US$ 25,000, another reporting US$ 25,000-US$ 49,999, and another reporting US$ 50,000 or more. Two informal caregivers indicated they had never been married, and 1 was currently married or widowed.

#### Clinicians

Thirty-seven clinicians working at the university where the research took place were eligible to serve as panelists, but 2 clinicians could not be reached via email. Of the 35 clinicians who were successfully contacted, only 2 (retention rate, RR, 6%) agreed to participate. All clinician panelists were men (mean age 36.50 years, SD 12.02 years). One clinician identified as being white, and the second clinician noted his race as “other.”

#### Research Scientists

Seventeen research scientists from the university where the research took place were eligible to serve as panelists, but 1 researcher could not be reached via email. Of the 16 researchers who were successfully contacted, 6 (RR 38%) agreed to participate. There were an equal number of female (n=3) and male (n=3) research scientists (mean age 38 years, SD 6.48 years). Research scientists identified as being white (n=5, 83%) and non-Hispanic (n=6, 100%).

#### Unidentified

Three panelists (8%) either did not want to disclose their stakeholder role or did not select one of the available stakeholder group options. Panelists who did not associate with one particular group reported a mean age of 50.33 years (SD 1.53 years), and identified their race/ethnicity as white (n=2, 33%), black or African American (n=1, 17%), Hispanic/Latino (n=1, 17%), other (n=1, 17%), or missing (n=1, 17%). All panelists in this group reported an annual household income less than US$ 25,000.

#### Study Drop Out

Eight panelists who completed the first-round survey did not complete the third-round survey. Study drop out was observed among all stakeholder groups: patients (n=2), informal caregivers (n=1), clinicians (n=1), and research scientists (n=2). A greater number of women (n=4) dropped out as compared with men (n=2). The highest dropout rate was observed among those who identified as white (n=5). Panelists reporting 1 year of college education or more (n=5) dropped out more often than panelists with other education levels. Most panelists who dropped out reported annual household incomes less than US$ 25,000 (n=6).

### Panelist Responses

#### First- and Second-Round Surveys

Of the 189 stakeholders representing all groups who were successfully contacted about participating on the virtual panel, 37 completed the first-round survey (RR 20%). Table 4 lists the mean (SD) ratings for each care coordination topic evaluated in the first and second rounds (1=strongly disagree, 7=strongly agree), with the corresponding percentage of panelists “agreeing” or “strongly agreeing” that the COPD care coordination topic was important to study using CER methods.

In the first-round survey, all care coordination topics received mean importance ratings of 6 or higher. “Measuring hospitalization risk,” received the highest mean rating (mean 6.55, SD 0.67), with over 90% of panelists (n=35) agreeing that this topic was important to investigate in future CER. Over 90% of the panelists also agreed that 4 other topics were especially important areas for future CER: “management of COPD with other chronic conditions” (n=35, mean 6.54, SD 0.61), “pulmonary rehabilitation as a model for care in COPD” (n=34, mean 6.47, SD 0.60), “quality of care coordination” (n=34, mean 6.54, SD 0.65), and “measurement of quality of care” (n=34, mean 6.43, SD 0.96). The care coordination topic that received the lowest mean rating was “patient-centered medical home” (mean 6.29, SD 1.00). As much as 72% of panelists (n=27) agreed that this topic was important to investigate.

Thirty-five of the 37 original panelists completed the second-round survey (RR 95%). As was the case in the first-round survey, all 10 care coordination topics received mean importance ratings above 6. In the second-round survey, “comprehensive COPD patient education” received the highest mean rating (mean 6.65, SD 0.81), with 91% of panelists (n=32/35) agreeing that this topic was important to investigate in future CER. Two other topics were also highly rated by a majority of panelists (≥88%; n=31/35): “pulmonary rehabilitation as a model for care in COPD” (mean 6.45, SD 1.12) and “quality of care coordination” (mean 6.39, SD 1.36). Interestingly, the topic, “management of COPD with other chronic conditions,” which was highly rated in the first round, scored lowest on perceived importance in the second round (mean 6.14, SD 1.44); however, over 85% of the panelists (n=30/35) agreed that this topic was important to study in future CER. Conversely, less than 80% of panelists agreed that “measuring hospitalization risk” (n=24/35) and “patient-centered medical home” (n=26/35) were important topics. Because consensus agreement was not reached on the importance of these 2 topics, panelists were not asked to evaluate either of these topics in the round-three survey.

**Table 4:** First- and second-round survey ratings for chronic obstructive pulmonary disease care coordination topics.

COPD care coordination topic^a^	Round one (N=37)		Round two (N=35)	
	Mean (SD)	Consensus agreement n (%)^b^	Mean (SD)	Consensus agreement n (%)^b^
Management of COPD with other conditions	6.54 (0.61)	35 (94)	6.14 (1.44)	30 (85)
Pulmonary rehabilitation	6.47 (0.60)	34 (91)	6.45 (1.12)	31 (88)
Depression and mental health management	6.38 (0.76)	33 (89)	6.44 (0.70)	30 (85)
Quality of care coordination	6.54 (0.65)	34 (91)	6.39 (1.36)	30 (85)
Measurement of quality of care	6.43 (0.96)	34 (91)	6.46 (0.78)	31 (88)
Comprehensive COPD patient education	6.50 (0.77)	32 (86)	6.65 (0.81)	32 (91)
Cost effectiveness of care	6.54 (0.78)	31 (83)	6.53 (0.71)	30 (85)
Case management	6.34 (0.84)	31 (83)	6.36 (1.14)	29 (82)
Measuring hospitalization risk	6.55 (0.67)	30 (81)	6.33 (0.89)	24 (68)
Patient-centered medical home	6.29 (1.00)	27 (72)	6.18 (1.07)	26 (74)

^a^Chronic obstructive pulmonary disease

^b^Consensus agreement was calculated by reporting the percentage of stakeholders who selected either 6 (agree) or 7 (strongly agree) on the 7-point Likert scale when evaluating each COPD care coordination topic.

#### Third-Round Survey

Twenty-nine participants completed the final third-round survey (RR 78%). Table 5 lists the mean (SD) importance rankings (1=most important, 8=least important) for the 8 remaining topics that received the greatest panelist support in the first 2 survey rounds. “Measurement of quality of care” (mean 2.73, SD 1.95) was ranked as the most important care coordination research topic to investigate in future CER. Other highly rated topics included the following (in order of importance with lower mean scores indicating greater importance): “management of COPD with other conditions” (mean 2.92, SD 1.67), “pulmonary rehabilitation as a model for care in COPD” (mean 3.73, SD 1.93), “quality of care coordination” (mean 4.12, SD 2.41), and “comprehensive COPD patient education” (mean 4.27, SD 2.38). Care coordination topics with the least amount of panelist support were “cost effectiveness of care” (mean 5.61, SD 2.33) and “case management” (mean 6.00, SD 2.38).

**Table 5:** Mean (SD) rankings of 8 remaining COPD care coordination topics (n=29).^a^

COPD^b^ care coordination topic	Mean (SD)
1. Measurement of quality of care	2.73 (1.95)
2. Management of COPD with other conditions	2.92 (1.67)
3. Pulmonary rehabilitation	3.73 (1.93)
4. Quality of care coordination	4.12 (2.41)
5. Comprehensive COPD patient education	4.27 (2.38)
6. Depression and mental health management	4.62 (2.04)
7. Cost effectiveness of care	5.61 (2.33)
8. Case management	6.00 (2.28)

^a^Topics were ranked from 1 (most important) to 8 (least important).

^b^Chronic obstructive pulmonary disease

Panelists provided 22 type-written comments that described reasons for their final rankings. Almost all of the comments (n=18/29, 62%) discussed the overall importance of strategies for COPD management. One panelist commented,

Given that comorbid conditions are commonly encountered together, along with a number of other chronic medical conditions and medications, clear management strategies in these types of populations are important.

Clinician, Male, Age 28

Patients described difficulty when attempting to manage medications intended to treat multiple health conditions. One patient stated,

I believe that if you manage this condition it will help overall with the other situations that one is faced with. I have thyroid disease along with vertigo and COPD. Having multiple conditions is very hard to manage; taking more than one medication and the different effects that they can have.

Patient, Female, Age 51

Panelists from almost all groups also commented on the need to determine the most effective ways to facilitate effective patient-provider communication. Miscommunication between stakeholder groups was cited frequently by panelists (n=20/29, 69%). One panelist who identified as both an informal caregiver and a clinician noted,

It has been my experience as an RN and family caregiver of my parents who both had COPD that there is a lack of communication between medical disciplines and grasp by the medical team of the overall health of COPD patients.

Informal Caregiver/Clinician, Female, Age 42

One research scientist also observed that

There are so many miscommunications between patients [with COPD] and providers.

Research Scientist, Male, Age 36

In addition, panelists noted the difficulty experienced by patients and providers in terms of identifying which patient education topics need to be covered with individual patients dealing with various stages of COPD. One panelist noted,

Although all the topics are very important for the care and results of the treatment of COPD, if a patient doesn't understand the basic importance of them, I don't believe they will be as effective. Especially the procedures.

Patient, Female, Age 61

When panelists were asked about the potential impact of a Web-based stakeholder network devoted to CER in COPD, they noted the importance of accessing results from recent CER studies (mean 2.34, SD 1.19). Patients, informal caregivers, and research scientists all indicated a strong desire to learn from others affected by COPD to build reciprocal trust and communication (mean 2.34, SD 1.19). Most panelists (n=22/29, 76%) also reported that they were likely to visit such a network website at least a few times/month. Panelists perceived their roles/responsibilities for network involvement to be primarily meeting with others interested in COPD (n=19/29, 66%), and suggesting, commenting on, and voting for new CER ideas in COPD (n=18/29, 62%). Finally, more than half of the panel noted the importance of accessing and learning from the following resources: COPD educational videos (n=24/29, 83%), news updates on CER taking place in COPD (n=18/29, 62%), and downloadable CER summaries (n=18/29, 62%). Patients and research scientists primarily requested access to educational videos on COPD care coordination, whereas clinicians preferred having Web access to recent research articles on CER in COPD.

## Discussion

### Principal Findings

The coordinating capacity of health care settings is often determined by the approaches and coordination activities used to deliver care. Achieving a good fit between coordination needs and coordinating capacity is key for effective and efficient care; however, the adequacy of fit is perceived differently by different stakeholders who are involved in processes of care at different levels. Traditionally, patients, informal caregivers, and health care providers have different perspectives on health care processes [56]. Yet, patients and informal caregivers are rarely included in the design of studies that test interventions or treatments. Paternalist approaches to research design and analysis often overlook important underrepresented groups who have increasingly requested that their voice be heard when conceptualizing CER relevant to health care problems they perceive to be important [57]. Because stakeholders from these groups are rarely afforded the opportunity to offer insight during both the decision- and priority-making processes for patient-centered health care [26], actively eliciting feedback from these stakeholder groups is becoming essential.

Because of this shift to patient-centered research designs, the primary purpose of this eDelphi study was to use a Web-based structured communication process to connect community-based stakeholders to discuss, generate, and evaluate potential COPD care coordination research topics that can be studied using CER methods. In this study, a team of faculty and clinical staff, in collaboration with research navigators working on behalf of a CEnR program, helped to organize a virtual advisory board consisting of stakeholders that represented 4 different stakeholder groups (patients, informal caregivers, clinicians, and research scientists). Results suggested that measuring quality of care delivered was the most important care coordination topic perceived by members of the virtual advisory board. Quality of care during the care transition process is especially critical for patients with COPD who are often treated for breathing exacerbations in the hospital or emergency room (ER). Often, these patients must proactively maintain pharmacotherapy and rehabilitation regimens based on changes in respiratory and physical condition [10]. In addition, quality of care measurement likely extends outside of primary care and into patients’ lives in the community and into linkages between medical practices and specialty clinics, ERs, and inpatient care settings [10]. Identifying measurement of quality of care as a high-priority COPD care coordination topic represents the first step in beginning a continuum of patient-centered outcomes research that will test the comparative effectiveness of alternative approaches to measuring care quality in this patient population. Further development of measurement models will require continued communication and input from stakeholders who will ultimately be affected by the findings from such CER.

The virtual advisory board further commented on the importance of disease management, communication, and education as key components to consider when assessing quality of care. For example, comprehensive patient education was noted to be tied to most all other care coordination topics evaluated. Collaborative patient education and stakeholder communication based on shared objectives were identified as critical to high-quality COPD care coordination both inside and outside of the clinical setting. Specifically, panelists commented on the need to evaluate different self-management education tools that patients could benefit from by successfully coping with the day-to-day challenges they encounter. Direct stakeholder feedback on these important needs can now be used as the basis for the development and evaluation of care-quality metrics assessing alternative COPD care coordination approaches used in this patient community.

Finally, to facilitate continued communication regarding research priority setting, a follow-up informal needs assessment was conducted following the main study to evaluate stakeholder interest in belonging, and contributing, to a collaborative Web-based community network for advancing CER in COPD. Findings suggested that the existence of a Web-based community would promote stakeholder understanding, engagement, and shared decision making, while building a new social media channel for generating, evaluating, and disseminating CER in COPD. Most panelists reported that they would be willing to regularly participate on a Web-based stakeholder engagement network to provide feedback on how to improve CER methods in COPD and review research summaries on the latest CER findings in COPD. This feedback will now be used to shape the purpose, scope, and function of the proposed Web-based stakeholder engagement infrastructure.

### Comparison With Prior Work

Previous research suggests that organizations often fail to capitalize on stakeholder engagement opportunities by only fostering one-way communication [58,59]. The current eDelphi study highlighted that user-friendly, survey technology can enable patients to become active contributors and partners throughout the CER process, especially during the development of study materials, data collection, and dissemination of research findings. Furthermore, the use of a Web-based stakeholder engagement platform helped to ensure that CER topics were communicated clearly and accurately in a manner conducive to understanding [60]. Results from this small-scale stakeholder engagement study shared some similarities with results of previous research conducted nationally in the United States. Krishnan and colleagues [20] reported the following COPD care coordination topics as being highly ranked by representatives of multiple stakeholder groups: (1) management of COPD with other chronic conditions; (2) pulmonary rehabilitation as a model for care in COPD; (3) depression and mental health management; (4) measurement of quality of care; and (5) quality of care coordination. In this eDelphi study, these topics were also rated highest in priority; however, measurement of quality of care was identified as the highest priority topic in this patient population. One exception was “depression and mental health management,” which was ranked just outside of the top 5 highest priority topics in this study. However, several panelists in this study commented on the worry and anxiety that patients experience due to their shortness of breath, which can often be mitigated by greater psychiatric care coordination and social support.

Similar to prior research [20], our prioritization study also identified “comprehensive COPD patient education” among the highest ranked topics. Many panelists submitted comments on the importance of patient education in care coordination for COPD. The need for comprehensive patient education resources that help patients and their informal caregivers manage the symptoms and treatments associated with obstructive lung disease has been emphasized in the literature. One qualitative study by Holland and colleagues [61] explored the content of education in pulmonary rehabilitation through semistructured interviews with patients diagnosed with interstitial lung disease. In their qualitative study, many patients emphasized the value of patient education, yet stated the education they generally received was often not applicable to their specific condition. Patients described the need for tailored symptom- and condition-specific education that was often missing in their treatment and action plans. Many panelists in our study confirmed that lack of communication is common among patients and providers, particularly with regard to access and use of inhalers and breathing medications.

### COPDFlix CER Network

**Figure 3:**
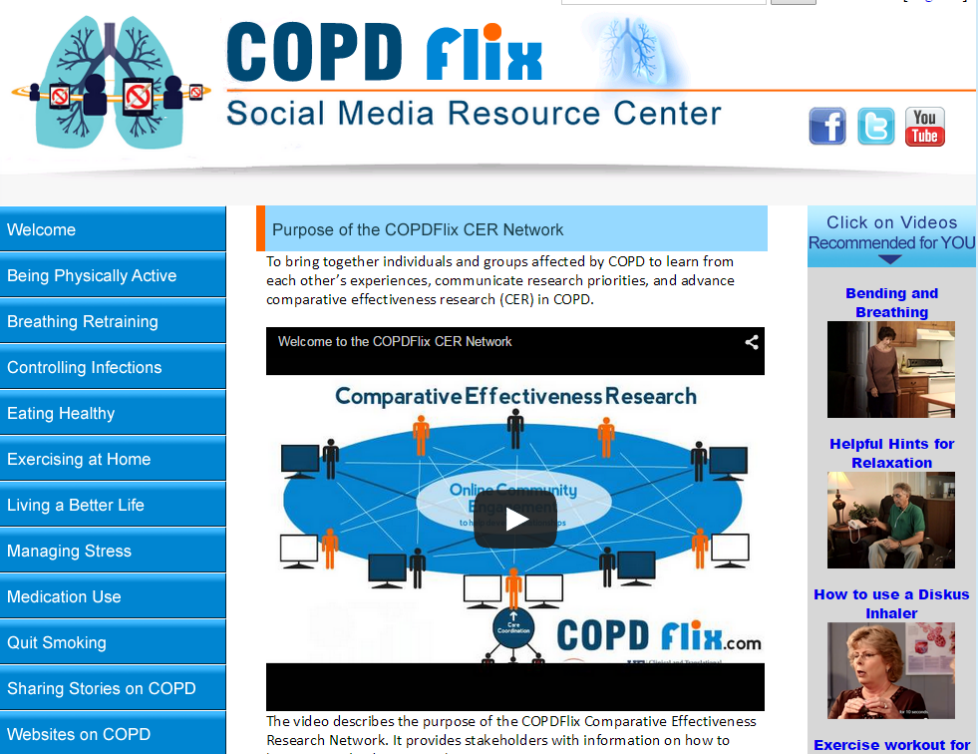
Screenshot of prototype version of the COPDFlix CER Network home page.

**Figure 4:**
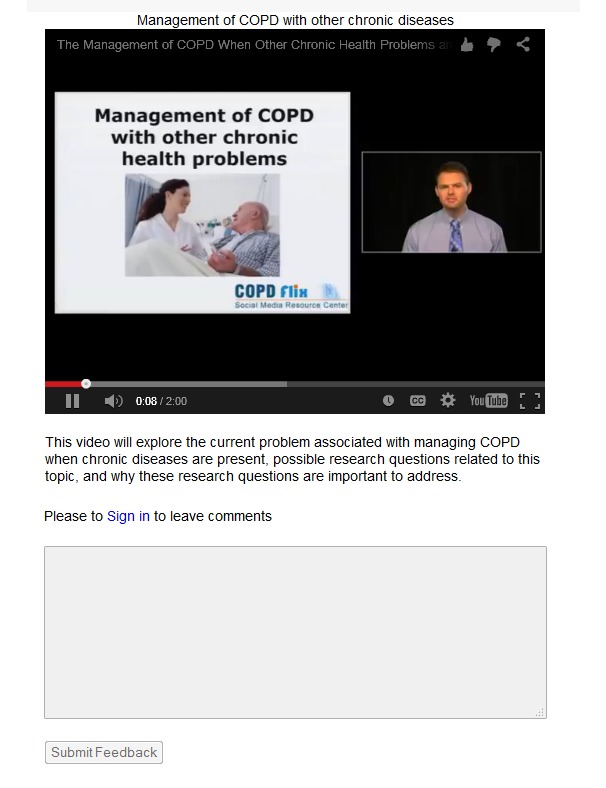
Screenshot of care coordination topic video and user comment box on COPDFlix CER Network page.

A recent systematic literature review by Moorhead and colleagues [62] suggests there are several overarching benefits to using social media to reach patients, health professionals, and the general public for health care purposes. These benefits include (1) increased interaction; (2) more information that is available, shared, and tailored; (3) greater health information accessibility; (4) enhanced instrumental support at the peer, social, and emotional levels; and (5) the potential to influence health policy. Through the utilization of CEnR methods, Stellefson and colleagues [63] consulted with experts in health ICT to create the COPDFlix Social Media Resource Center, which was codesigned with medically underserved patients living with COPD, who experience low computer literacy [64]. The purpose of this Web-based stakeholder engagement network is to disseminate knowledge on COPD self-management, and strengthen COPD research collaborations at multiple ecological levels [65]. Using the feedback received from stakeholders during this eDelphi study, our research team built on the existing COPDFlix social media website to create the technical infrastructure for the COPDFlix CER Network. It is expected that this additional Web-based community engagement center will include CER collaborations in COPD with diverse stakeholders including patients and informal caregivers. At present, the site displays a stakeholder-orientation video describing the intended purpose of the network (Figure 3) along with selected COPD care coordination/CER videos evaluated by the virtual advisory board members in this study (Figure 4).

On the network home page, users can click on videos uploaded using the YouTube media player that describe the 5 most important care coordination topics as rated by panelists in our study. Users are also able to post and respond to comments regarding the video content using textboxes attached to each video. Figure 4 shows a screenshot of one of the care coordination topic videos that users can rate (like/dislike), share, and comment on by signing into the network home page. Based on suggestions provided by the virtual advisory board, the COPDFlix CER Network is being used to post relevant educational videos, research articles, and news reports on CER in COPD. By uploading discrete videos on various care coordination topics, we foresee opportunities for continued stakeholder engagement around selected CER questions of interest. Further development of the COPDFlix CER Network will enable researchers to post available research opportunities, involve representatives from multiple stakeholder groups, and disseminate results from CER studies in COPD to the public at-large. Translating results from this study into the development of specific study designs and research proposals is an important next step to advance patient-centered CER in COPD.

### Limitations

Low initial response rate was one limitation of this study, with only 37 of 189 contacted (20%) stakeholders participating on the virtual panel. Reasons for the low response rate included the following: (1) lack of patient access to a computer; (2) low knowledge on how to use a computer; and (3) time constraints. While the use of only 1 CEnR program for recruitment may have limited the total number of respondents, the focus of this study was on gathering opinions from community-based stakeholders who could potentially be involved with the development and evaluation of CER proposals in COPD. Adding a paper-based alternative for the round-by-round questionnaires may have increased the response rate from patients and informal caregivers. In addition, the use of Web-based surveys may have limited the generalizability of findings to only those medically underserved patients with computer access and experience. However, it is important to note that several patients were able to travel to the CEnR program headquarters and complete the Web-based survey in their computer laboratory with assistance from program staff. The low initial response rate for this research priority-setting study highlights the need for future Web-based stakeholder engagement research within low SES populations. This type of research would benefit from incorporating computer and Internet literacy trainings prior to and during all research-related activities.

Few informal caregivers (n=3) were enrolled into the study at any point in time, which we attributed, in part, to the snowball sampling referral method used to recruit stakeholders from this group. Informal caregivers could not be contacted directly by research navigators affiliated with the CEnR program, because of patient privacy risks and the potential for informal caregivers feeling coaxed into participation. In addition, several individuals living with COPD felt that they did not need assistance from a family member or friend to manage their COPD, and therefore, they reported having no informal caregivers. Future studies seeking input from informal caregivers may benefit from using alternative sampling methods and seeking out members from informal caregiver registries operated by local/regional health care agencies and organizations.

Similarly, few eligible research scientists (n=6) agreed to participate on the virtual panel. Initial panel recruitment occurred primarily in the summer months, which may have conflicted with the regular academic calendar (fall/spring) for some researchers. Clinicians were the least represented stakeholder group on the panel, with only 2 of the 35 clinicians agreeing to serve as a panelist. Potential reasons for the very low response rate among clinicians may have included lack of time, interest, and limited incentive for participation (US$ 45 gift card). Because of the nonrandom and very small samples in several stakeholder groups, findings from this study may not be representative and generalizable to the populations they represent. Nevertheless, the purpose of this Web-based stakeholder engagement study was to establish the validity and reliability of using the eDelphi technique to generate CER priorities in a community where previous research has not been conducted, and therefore, our methodological emphasis was appropriately on maximizing internal validity versus establishing external generalizability. Future stakeholder consensus-building effort may benefit from investing more time developing collaborative relationships with key clinician/research scientist gatekeepers (eg, leadership of local medical societies, senior clinicians, chairs of respiratory health departments) prior to study recruitment. Further development of our COPDFlix CER Network is expected to improve stakeholder recruitment effort in future CER studies.

Finally, the expertise of eDelphi panelists (patients, informal caregivers, clinicians, and research scientists) was not uniform. Stakeholders from different groups likely possessed different levels of knowledge regarding CER and COPD care coordination. Some panelists chose not to identify their stakeholder group affiliation, because they may have felt uncomfortable providing this information in this university-based study. There was also potential for intellectual, financial, and clinical conflicts of interest, which may have biased rankings, especially among research scientists and clinicians. To our knowledge, and as acknowledged by other researchers [20], no standards exist for managing and disclosing potential conflicts of interest when setting CER priorities. The eDelphi technique may be subject to both researcher and subject biases because panelists may change their own opinions to fit their own personal, research, and clinical agendas. Notwithstanding this potential, the collaborative aspect of the eDelphi technique supports acknowledging and considering the perspectives of others to achieve meaningful consensus, which is considered a key strength of this structured communication process [30].

### Conclusions

Although engaging representatives of diverse stakeholder groups is feasible and can be used to identify support for CER topics in COPD [20], engaging individuals who actually belong to important stakeholder groups is sometimes overlooked. Patients from medically underserved communities are rarely afforded the opportunity to provide feedback on compliance with intervention protocols and experimental treatments**.** This study effectively used the eDelphi method to engage medically underserved patients to identify community-specific priorities in COPD care coordination. The inclusion of low-income, traditionally underrepresented patients with COPD provided a myriad of perspectives, which helped to identify care coordination topics that may have been overlooked with a typical homogeneous panel. Identification of measurement of quality of care coordination should be viewed as a preliminary finding to structure both Web-based and offline discussions regarding future CER proposals/protocols. Moreover, future CER in COPD care coordination should incorporate measurement mechanisms for care coordination activities and approaches, which can positively impact patients, their families, health care professionals, and the overall health care system.

Effectively engaging patients with COPD from various racial, ethnic, aged, and low-resourced backgrounds is important during all stages of the CER process (eg, planning, data collection, choice of intervention, analysis of results, and implementation of findings into clinical practice). Findings from this study support the need to conduct more CER that explores whether the use of eHealth and social media represents an acceptable and effective way to engage low-income and racial/ethnic minority populations for CER in COPD. Web-based stakeholder networks have the potential to engage more stakeholders and improve the study of COPD care coordination using CER methods. Future studies would benefit from collecting more robust data from larger, more representative samples of community-dwelling stakeholders interested in improving COPD care.

## Abbreviations

CEnR: community engagement and research
CER: comparative effectiveness research
COPD: chronic obstructive pulmonary disease
ER: emergency room
ICTs: information and communication technologies
RR: retention rate
SES: socioeconomic status
